# A biolistic method for high-throughput production of transgenic wheat plants with single gene insertions

**DOI:** 10.1186/s12870-018-1326-1

**Published:** 2018-06-26

**Authors:** Ainur Ismagul, Nannan Yang, Elina Maltseva, Gulnur Iskakova, Inna Mazonka, Yuri Skiba, Huihui Bi, Serik Eliby, Satyvaldy Jatayev, Yuri Shavrukov, Nikolai Borisjuk, Peter Langridge

**Affiliations:** 1 School of Agriculture, Food and Wine, University of Adelaide, Urrbrae, SA 5064 Australia; 2 Present address: NSW Department of Primary Industries, Wagga Wagga Agricultural Institute, Pine Gully Road, Wagga Wagga, NSW 2650 Australia; 3 Present address: Aytkhozhin Institute of Molecular Biology and Biochemistry, Almaty, 480012 Kazakhstan; 4 Present address: National Key Laboratory of Wheat and Maize Crop Science, Henan Agricultural University, Zhengzhou, 450002 China; 5 S.Seifullin Kazakh AgroTechnical University, Astana, 010011 Kazakhstan; 6 College of Science and Engineering, School of Biological Sciences, Flinders University, Bedford Park, SA 5042 Australia; 7 Present address: School of Life Science, Huaiyin Normal University, Huaian, 223300 China

**Keywords:** Biolistic transformation, DNA/gold coating, Immature embryo, Single copy events, Transformation frequency, *Triticum aestivum*

## Abstract

**Background:**

The relatively low efficiency of biolistic transformation and subsequent integration of multiple copies of the introduced gene/s significantly complicate the genetic modification of wheat (*Triticum aestivum*) and other plant species. One of the key factors contributing to the reproducibility of this method is the uniformity of the DNA/gold suspension, which is dependent on the coating procedure employed. It was also shown recently that the relative frequency of single copy transgene inserts could be increased through the use of nanogram quantities of the DNA during coating.

**Results:**

A simplified DNA/gold coating method was developed to produce fertile transgenic plants, via microprojectile bombardment of callus cultures induced from immature embryos. In this method, polyethyleneglycol (PEG) and magnesium salt solutions were utilized in place of the spermidine and calcium chloride of the standard coating method, to precipitate the DNA onto gold microparticles. The prepared microparticles were used to generate transgenics from callus cultures of commercial bread wheat cv. Gladius resulting in an average transformation frequency of 9.9%. To increase the occurrence of low transgene copy number events, nanogram amounts of the minimal expression cassettes containing the gene of interest and the *hpt* gene were used for co-transformation. A total of 1538 transgenic wheat events were generated from 15,496 embryos across 19 independent experiments. The variation of single copy insert frequencies ranged from 16.1 to 73.5% in the transgenic wheat plants, which compares favourably to published results.

**Conclusions:**

The DNA/gold coating procedure presented here allows efficient, large scale transformation of wheat. The use of nanogram amounts of vector DNA improves the frequency of single copy transgene inserts in transgenic wheat plants.

## Background

Plant genetic transformation using biolistic microprojectile bombardment is broadly applied in the generation of transgenic plants, including important cereal crops, such as rice (*Oryza sativa*), maize (*Zea mays*) and wheat (*Triticum aestivum*) [1–3]. As one of the main industrial crops, wheat has attracted considerable attention in the establishment and optimization of efficient transformation methods [3–15]. In most of these publications, the DNA coating preparation remained largely unchanged from the original protocol based on the formation of an unstable DNA/spermidine/Ca^2+^ complex [16]. Despite some optimization [17, 18], the reproducibility of this method remains quite variable in large-scale stable transformation experiments.

Another major concern of stable biolistic transformation is random, multi-copy transgene integration, commonly resulting in irregular compositions of inverted repeats or transgene rearrangements. These can lead to transgene silencing [19, 20], aberrant transgene expression in subsequent generations [21, 22] and even loss of the transgenes [23]. Transgenic plants with single/low copy inserts are less affected by these problems [24], and thus are desirable for functional genomic studies or the production of marker free plants after genetic segregation. Using minimal transgene cassettes instead of bombarding with whole plasmid DNA can increase the transformation frequency (TF) with simple transgene integration patterns [13, 25, 26]. To date, this strategy has been applied in the transformation of several monocotyledonous species such as wheat [13], maize [26], pearl millet (*Pennisetum glaucum*) [27], and sugarcane (*Saccharum officinarum*) [28]. Furthermore, low transgene copy number is found to be inversely correlated with the amount of the DNA cassette used in the biolistic transformation [26]. Quantities as low as 2.50–2.73 ng of DNA cassette per shot have been recommended for the efficient production of single copy events in maize [26] and in sugarcane [29].

In this paper, we present a biolistic transformation method that uses a reduced load of DNA expression cassettes in combination with the microprojectile particles coating procedure based on PEG/MgCl_2_. This process results in relatively high transformation frequency and greater representation of single transgene copy events, averaging 38.2% as deduced from the examination of 1538 transformed wheat plants in 19 independent experiments.

## Methods

### Plant material and explants

The spring wheat cv. Gladius was utilised throughout this research. Gladius is an elite Australian cultivar with the pedigree RAC875/Krichauff//Excalibur/Kukri/3/RAC875/Krichauff/4/RAC875//Excalibur/Kukri. Donor plants were maintained in a glasshouse (23 °C, 16/8 h day/night). Immature grains were collected 11–14 days after flowering and surface-sterilized in 70% (v/v) ethanol for 2 min, then for 20 min in 1% NaOCl (sodium hypochlorite), followed by three rinses in sterile water. The excised embryos were incubated, scutellum side up, on solidified callus induction MS [30] medium containing 500 mg/L casein hydrolysate, 30 mg/L CP (centrophenoxine), 30% sucrose, and 5 g/L gelrite (MS30-CP) for 7–14 days before transformation [15]. *T. monococcum* L. suspension cells [31] were propagated in 250 ml flasks with weekly subcultures in liquid basal MS2–2,4D medium with 2 mg/L 2,4D and 30 g/L sucrose.

### DNA constructs used in transformation

**Fig. 1:**
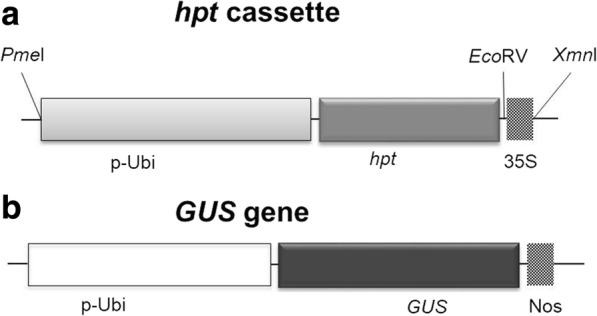
Schematic representation of the *hpt* and *GUS* gene constructs used in the transformation experiments. P-Ubi, ubiquitin promoter (1.6 Kb); *hpt*, hygromycin resistance gene (1 Kb); *GUS*, *gusA* gene (1.8 Kb); 35S, 35S terminator (200 bp); Nos, Nos terminator (258 bp). About ~ 3 Kb *hpt* DNA cassette was used for stable transformation

The *GUS* (*β-*glucuronidase A) gene driven by the P-Ubi promoter (1986 bp) [32] was used for transient expression analysis (Fig. 1). For stable transformation, the ~ 3.0 Kb selectable marker DNA cassette, containing the P-Ubi promoter, *hpt*, hygromycin resistance gene, and cauliflower mosaic virus 35S Terminator, was cut out with *Pme*I and *Xmn*I restriction enzymes (New England Biolabs) (Fig. 1). Plasmids carrying the Genes of Interest (GOI), represented by 19 new wheat genes isolated at the University of Adelaide, were digested with restriction enzymes to release the DNA cassettes, and isolated by electrophoresis on 1% agarose gels. The gel pieces bearing the minimal DNA cassettes were processed with the GenElute Gel Extraction Kit (Sigma Aldrich).

### DNA/gold coating procedure

The DNA/gold coating procedure corresponding to the initial Bio-Rad protocol [16] was carried out as follows: 50 μl aliquots of gold microparticles (0.6 μm, 30 mg/ml) suspended in 50% (v/v) sterile glycerol solution were combined with DNA (5 μg in 5 μl of sterile water), 2.5 M CaCl_2_ (50 μl) and 0.1 M spermidine, Spd (20 μl) under constant vortexing. After vortexing for several minutes, the suspension was incubated at room temperature for 20 min. The coated DNA was then pelleted by 1 min centrifugation at 3920×*g*. The pelleted DNA was washed with 100 μl of 70% (v/v) ethanol, followed by 100 μl of absolute ethanol and finally resuspended in 60 μl of absolute ethanol. Six microliter aliquots of the coated DNA were used per bombardment.

The PEG/Mg^2+^ (PM) coating procedure was executed as follows: 50 μl aliquots of gold microparticles were combined with DNA (10 μl) and supplemented under vortexing with 10 μl of PM solution (42% PEG 2000 and 560 mM MgCl_2_). The stock solutions of 52.5% PEG 2000 (Sigma-Aldrich) and 2.8 M MgCl_2_ (filter sterilized) were mixed at a ratio of 4:1. The suspension was vortexed for 1 min, followed by 20 min incubation at room temperature and a 1–5 min centrifugation (longer for diluted DNA) to pellet the coated DNA. The pelleted DNA was washed once with 100 μl of absolute ethanol and resuspended in 60 μl of absolute ethanol (6 μl per bombardment). All steps were carried out at room temperature. In stable transformation experiments, 60 ng of selective marker gene (Fig. 1a) and 60 ng of GOI DNA cassettes were used for DNA/gold coating (50 μl gold + 10 μl of DNA + 10 μl PM solution).

### Plant transformation

Particle bombardment was conducted using the Particle Delivery System PDS-1000/He (Bio-Rad). Embryogenic callus was osmotically pre-treated on MS5-CP medium [15] containing 100 g/L sucrose for 4 h. Callus pieces (ca. 1–2 mm) placed in the centre of the plate to form a circle with a diameter of 25–30 mm were bombarded at 900 psi, with the 15 mm flight distance and a 60 mm target distance. The bombarded calli were transferred to MS5-CP medium 16 h after the treatment and were grown in the dark for 1 week. Plantlet selection and regeneration procedures were as described earlier [15].

### Transient GUS assay

The uncut plasmid DNA pAHC25 [32] was used only for transient expression analysis in suspension cells which were treated 4 days after subculture. Five ml of well-mixed suspension adjusted to OD = 0.1–0.2 was spread on Ruled Qualitative Filter Paper discs with a 5 mm Ruled Grid, diameter 55 mm (Whatman, USA). A vacuum was applied to get an even spread of a very thin layer of cells on the surface of the filter paper. The coated papers were subsequently transferred to MS2–2,4D medium with 100 g/L sucrose for osmotic pre-treatment for 4 h. Bombardment conditions for the callus tissues and the suspension cells were the same as described above. The transformed cells were maintained on the MS2 medium for 48 h. GUS staining with X-gluc (0.5 mg/mL) was as described in [33]. Filter disks with the treated cells were carefully transferred into 60 mm plastic Petri dishes with 550 μl of the X-gluc solution and incubated overnight at 37 °C. For further details see [34].

To quantify GUS expression in the *T. monococcum* suspension cells, the number of cells with blue staining was counted on the same Ruled Qualitative Filter Papers under a Binocular microscope (Nikon, Japan), and recorded as described earlier [34, 35]. Each treatment was repeated at least three times, with mean and variability calculated.

### DNA isolation, qPCR analysis and Southern hybridisation

DNA was extracted from leaf tissue using the freeze-dry method for qPCR analysis [36] and using the phenol-chloroform method for the Southern hybridisation [37]. Total DNA was quantified using a Nano-Drop spectrophotometer (ND-2000, NanoDrop Technologies Inc., USA). Transgene copy numbers were determined by Real-time quantitative PCR using two reference genes, Cyclophilin and Glyceraldehyde-3-phosphate dehydrogenase, as described in [38], and confirmed with a cross-test of the same individuals by Southern autoradiography hybridisation. For the Southern analysis, DNA samples were digested with *Eco*RV at 37 °C for 5 h, run on a 1.0% (v/v) agarose gel, blotted onto a Hybond N^+^ nylon membrane (GE Healthcare Life Sciences, NSW, Australia) and subjected to hybridisation with a ^32^P-labelled DNA probe specific to the transgene following standard methods. The DNA probe was labelled using the random primer method with the Ready-to-Go DNA labelling kit beads (GE Healthcare Life Sciences, NSW, Australia). An ‘in-house’ probe (35S, 354 bp) targeting part of the backbone of the 4237 bp transformed DNA fragment was used. Primers for amplifying this probe by PCR were as follows: F: 5’-CAACATGGTGGAGCACGAC-3′ and R: 5-GCGTCATCCCTTACGTCAGTGGAG-3′.

### Statistical analysis

Mean and standard errors were calculated with ANOVA using standard Excel software. Probabilities for significance were calculated using Student’s ***t***-test.

## Results

### PEG/Mg^2+^ coating procedure

**Fig. 2:**
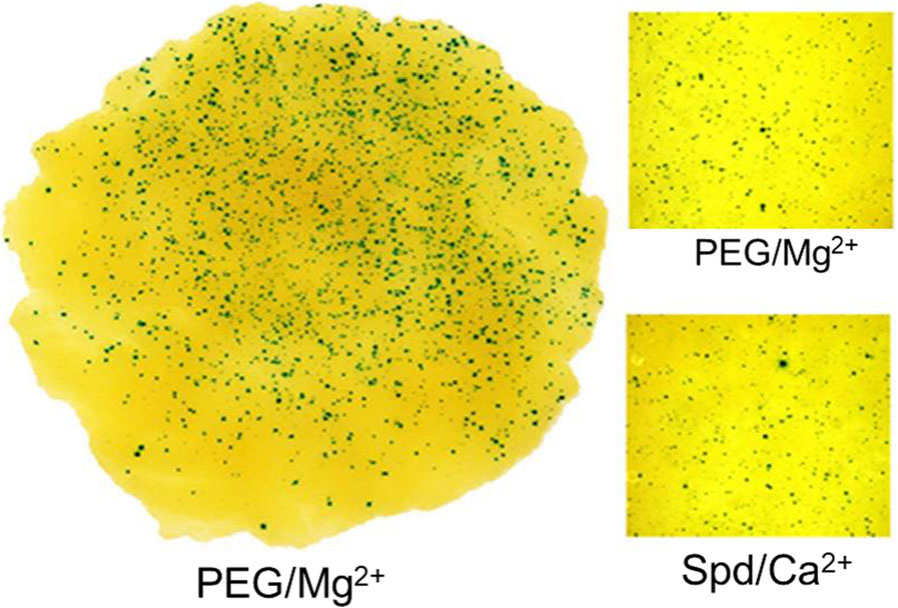
The bombarded *T. monococcum* suspension cells. The treated cultures exhibit relatively uniform distribution of the X-Gluc stained cells over an area of 30–40 mm in diameter

**Fig. 3:**
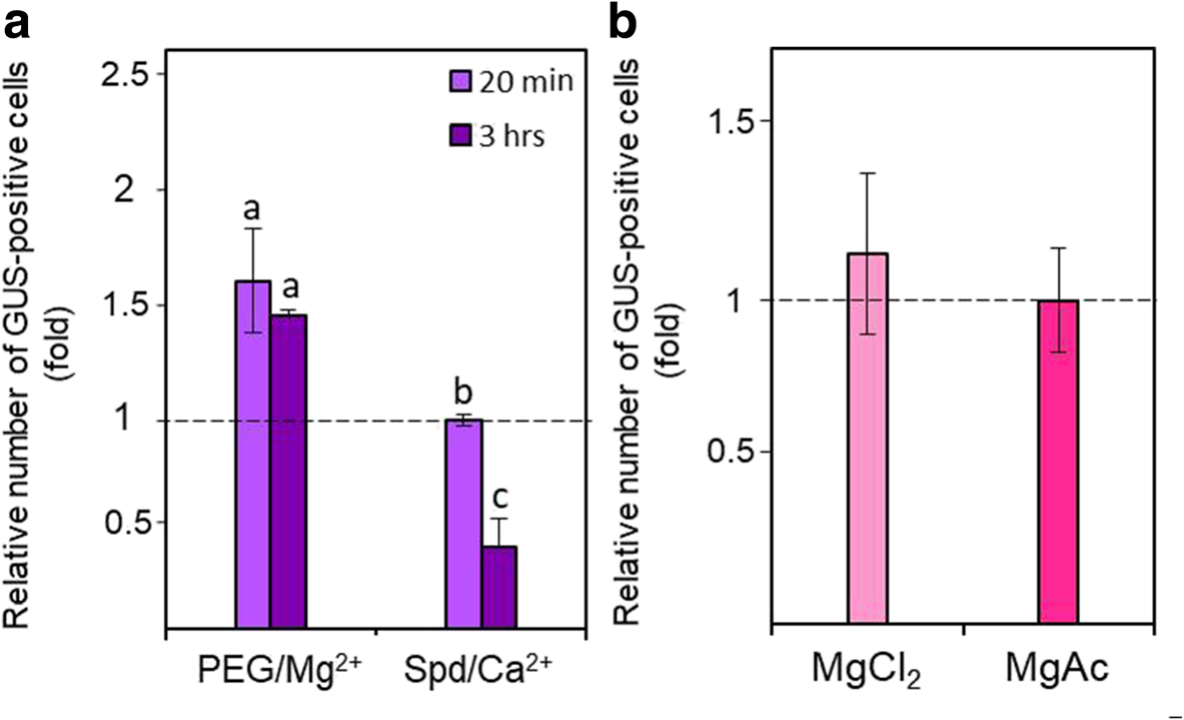
Relative number of GUS-positive cells in wheat cell suspension cultures bombarded under different DNA/gold coating conditions. **a** Incubation time (20 min and 3 h, light and dark purple, respectively) for different coating methods. **b** Sources of ions: 80 mM MgCl_2_ or MgAc. P-Ubi:GUS DNA cassette (300 ng per shot) was used for each treatment. The Spd/Ca^2+^ method with 20 min incubation was set as one unit. Bars represent means ± standard errors for three replicates. Different letters above the bars represent significant differences (*P* < 0.05) using Student’s *t*-test

PEG and Mg^2+^ solutions are known to allow efficient DNA precipitation at room temperature [39, 40]. We found that they can be applied successfully in DNA/gold coating (Fig. 2). In transient transformation experiments with 6% PEG / 80 mM MgCl_2_, the relative numbers of GUS-positive cells were, on average, between 1.2-fold [41] and 1.6-fold (Fig. 3a) higher compared to the Spd/CaCl_2_ coating method. It was also found that the PEG/Mg^2+^ DNA/gold coating mixture remains relatively stable for 3 h at room temperature providing for additional flexibility in long experiments and implying that this method offers better DNA protection. The GUS transient expression, based on relative numbers of GUS-positive cells, was lower by 3.9-fold after 3 h of incubation at room temperature for the original Spd/Ca^2+^ coating mixture (Fig. 3a). Magnesium chloride (MgCl_2_, pH = 5.5) and magnesium acetate (MgAc, pH = 6.5) at 80 mM concentration had a comparable effect on the successful DNA/gold co-precipitation although there was an average of 1.14-fold improvement with MgCl_2_ compared to MgAc (Fig. 3b). Therefore, in the stable transformation experiments, PEG-MgCl_2_ was used for coating as the optimal method.

### Efficient regeneration of single copy T_0_ transgenic wheat plants

To obtain high numbers of single copy events, a ratio of 1:1 of 6 ng/per shot each of the GOI and *hpt* cassette was used for the large-scale generation of transgenic wheat plants. In total, we isolated 15,496 immature embryos for 19 independent biolistic transformation experiments (Table 1). The average transformation frequency was 7.4% for GOI and 9.9% (between 3.1 and 20.3%) for *hpt* with a total of 1538 hygromycin resistant transgenic plants.

**Table 1:** Summary of T_0_ transgenic wheat plants regenerated in 19 independent events of biolistic transformations

Experiment	No. trans-formed embryos	Transgene copy No.				No. hygromycin resistant plants (HmR)	No. GOI transformants	GOI with one copy transformants (%)	Transformation frequency, TF, HmR (%)	Co-transformation, Co-TF (%)
		0	1	2	≥3					
T01	857	18	19	4	17	58	40	47.5	6.8	69.0
T02	998	36	29	7	40	112	76	38.2	11.2	67.9
T03	1005	21	22	5	29	77	56	39.3	7.7	72.7
T04	912	21	20	2	56	99	78	25.6	10.8	78.8
T05	899	14	23	7	44	88	74	31.1	9.8	84.1
T06	753	18	15	0	13	46	28	53.6	6.1	60.9
T07	950	37	60	31	65	193	156	38.5	20.3	80.8
T08	914	62	48	14	28	152	90	53.3	16.6	59.2
T09	936	38	51	16	6	111	73	69.9	11.9	65.8
T10	735	10	25	14	38	87	77	32.5	11.8	88.5
T11	729	20	19	7	82	128	108	17.6	17.6	84.4
T12	662	14	25	8	1	48	34	73.5	7.2	70.8
T13	620	2	15	6	72	95	93	16.1	15.3	97.9
T14	708	11	30	2	38	81	70	42.9	11.4	84.6
T15	847	33	6	2	7	48	15	40.0	5.7	31.3
T16	856	16	8	5	7	36	20	40.0	4.2	55.6
T17	777	5	8	4	7	24	19	42.1	3.1	79.2
T18	681	1	7	4	10	22	21	33.3	3.2	94.5
T19	657	12	9	1	11	33	21	42.9	5.0	63.6
Total	15,496	389	439	139	571	1538	1149			
Transformation, %	100					9.9	7.4			74.7
GOI transformation, %		–	38.2	12.1	49.7		100			

Data summarized for 19 stable transformation experiments. The transgene copy numbers of GOI in each event were determined by qPCR and confirmed by the Southern blot

**Fig. 4:**
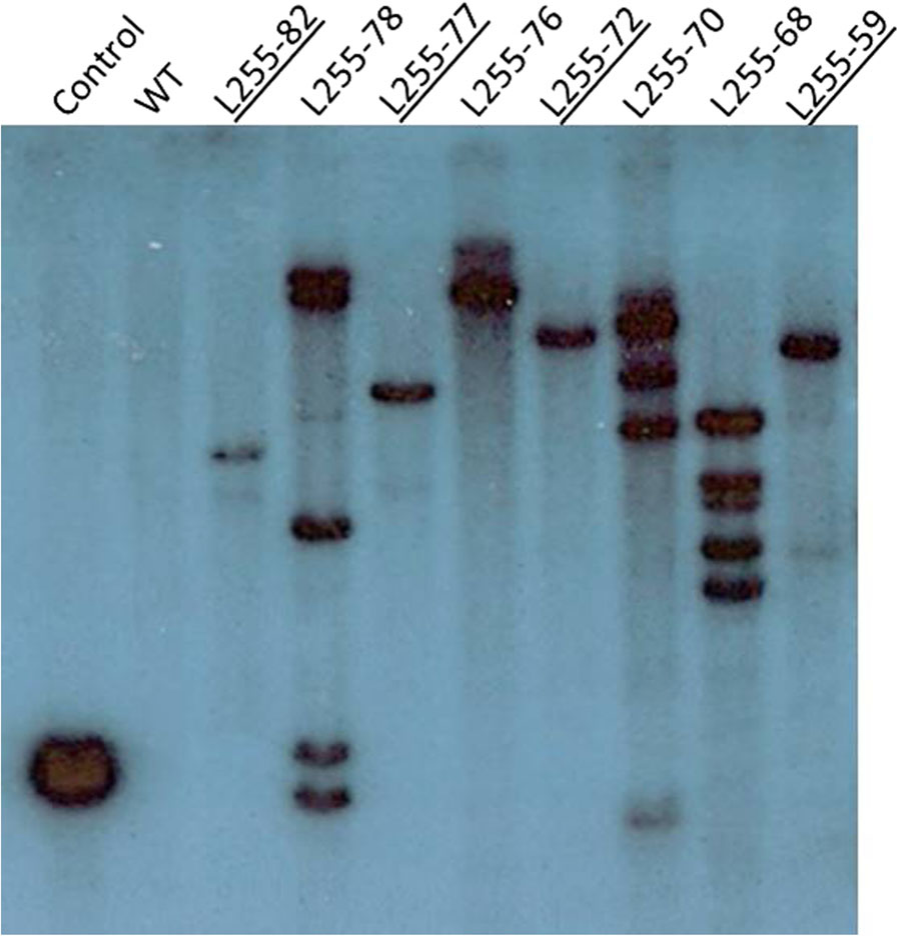
Example of Southern blot of transgenic wheat plants with different copy numbers of the transgene. Four out of the eight transgenic lines had single transgene insertions (underlined). Four other transgenic lines (not underlined) had multiple copy numbers of the transgene. A positive control, consisting of DNA from a barley plant with a single transgene insertion is indicated as the ‘Control’. The negative control is represented by wild-type (WT) wheat DNA (cv. Gladius)

The transgene copy numbers in each T_0_ plant were determined by qPCR and confirmed with a cross-examination of the same individuals using the Southern hybridisation. The example of the Southern blot is present in Fig. 4, where four out of eight T_0_ transgenic plants showed unique pattern of a single copy of the transgene insertion.

**Fig. 5:**
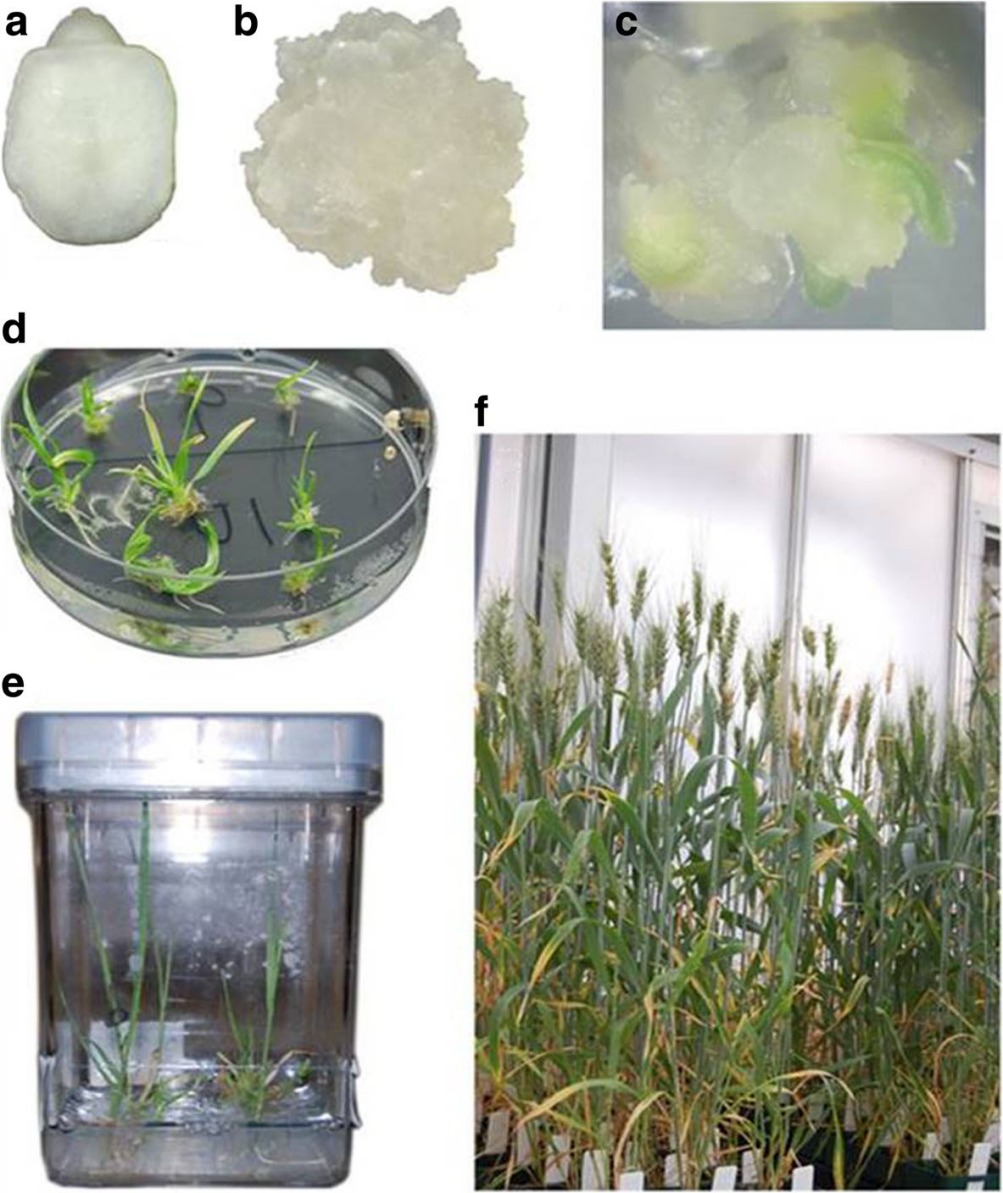
Biolistic wheat transformation process with hygromycin selection. **a** Freshly isolated immature embryo; (**b**) 2 week-old embryogenic callus before bombardment; (**c**) Shoot regeneration on 50 mg/L hygromycin selection medium; (**d**) Shoot and root regeneration on 50 mg/L hygromycin selection medium; (**e**) Plantlet recovery in 1/2 MS medium; (**f**) T_0_ transgenic plants in the greenhouse

Of all the transgenic plants, the frequency of single copy events varied from 16.1% in T13 to 73.5% in T12 with an average of 38.2%. In experiment T07, the number of single copy events reached 60, in comparison to 15 in experiments T06 and T13. Interestingly, the total number of two copy events was 139, which was three-fold lower than the number of single copy events (439). The in vitro tissue culture period from the isolation of embryos to the regeneration of transgenic plants was between 140 and 190 days (Fig. 5).

## Discussion

The PEG/Mg^2+^ coating procedure in the current study showed improved results compared to the standard Spd/Ca^2+^ protocol in transient transformation. The Bio-Rad coating method relies on high quality spermidine which can differ from batch to batch. Spermidine solutions are hydroscopic, oxidisable, deaminated with time and, therefore, the frozen aliquots should be made fresh at least once a month [16, 20]. The stock solutions of CaCl_2_ and spermidine cannot be combined together in one solution and must be used separately. In contrast, PEG and magnesium salts can be conveniently made in a single stock solution that remains stable for years when stored at − 20 °C. Gold/DNA coating in transformation experiments is done by simple mixing 50 μl gold suspension with 10 μl DNA and 10 μl of PM solution under vortexing at room temperature. To date, the PEG/Mg^2+^ procedure described here has been applied successfully in stable transformation of spring wheat cvs. Bobwhite, Akadaruma [41] and Gladius using embryogenic callus cultures as well as in producing transgenic winter wheat, durum wheat, maize, sorghum, pearl millet and eastern gamagrass (data not shown).

A study in rice found that 80% of transgenic lines transformed with minimal cassette DNA showed simple integration patterns, compared with 20–30% of lines transformed with plasmid DNA [25]. The use of minimal DNA cassettes instead of plasmid DNA in biolistic transformation almost tripled the frequency of wheat stable transformation from 0.4 to 1.1% [42]. Furthermore, when nanogram amounts of DNA cassette were employed (25 ng and 2.5 ng per shot [26], the regeneration of low copy transgenic maize plants became more frequent. In sugarcane, bombardment with 6.6 ng per shot of the minimal cassettes resulted in over 30% of low copy integration events [28]. In the present study, co-transformation frequencies (co-TFs) of the GOI and selective marker minimal expression cassettes were between 31.3 and 97.9% with an average of 74.7%, which is comparable to the results in sugarcane using 6.6 ng of DNA/shot (79–84%) [28] and in wheat using 200 ng of DNA/shot (91.7%) [42]. The large difference in co-TFs in our experiments is likely due to the impact of various GOIs on co-integration and plant regeneration.

The efficiency of wheat biolistic transformation remains relatively low at 1–5% [43]. In recent publications, the use of 5 ng per shot of the expression cassettes in the model wheat cv. Bobwhite S26 resulted in 50–60% of transformants with simple integration pattern at a TF of 2.7–4.0% [44]. In our experiments with the commercial wheat cv. Gladius, the TFs ranged from 3.1 to 20.3%. TFs below 5% were observed only in three experiments (T16-T18) out of 19. Variations in TFs between experiments can be attributed to seasonal fluctuations in the quality of immature embryos, to GOI effects on plant transformation, to changes in tissue culture conditions due to batch-to-batch differences in reagent quality, etc.

We produced between 16.1–73.5% single copy events (Table 1) by using 6 ng of minimal GOI DNA cassettes. This is comparable to the 41–80% reported in transformation of maize (2.5 ng DNA cassette per shot) [45], and to the 49.2% for sugarcane transformation (2.73 ng DNA cassette per shot) [29]. The results suggest that the use of nanogram amounts of DNA cassette is desirable when seeking to regenerate plants with low copy insertion of the transgene. However, the optimal quantity for each plant species in biolistic transformation needs more investigation [46].

## Conclusions

The use of PEG and Mg^2+^ instead of Spd and Ca^2+^ in the conventional biolistic coating procedure was developed to transform wheat and other plant species in both transient and stable transformation. In bombarding a low quantity of minimal DNA cassette was reported to efficiently regenerate low copy transgenic plants. Nanogram amounts of the minimal expression cassettes of the GOI and the *hpt* gene were routinely used in high-throughput experiments to generate single copy transgenic plants of commercial wheat at a high frequency.

## Abbreviations

CoTF: Co-transformation frequency
CP: Centrophenoxine
GOI: Gene of Interest
*GUS*: *β-glucuronidase A* gene
*Hpt*: Hygromycin resistance gene
MS: Murashige and Skoog medium
PEG: Polyethyleneglycol
psi: Pounds per square inch, a unit of pressure
Spd: Spermidine
TF: Transformation frequency
X-gluc: 5-Bromo-4-chloro-3-indolyl-β-D-glucuronide, a reagent to detect *β-*glucuronidase
